# Efficacy of oat hulls varying in particle size in mitigating performance deterioration in broilers fed low-density crude protein diets

**DOI:** 10.1016/j.psj.2023.102979

**Published:** 2023-07-28

**Authors:** M. Naeem, E. Burton, D. Scholey, A. Alkhtib, S. Broadberry

**Affiliations:** School of Animal, Rural and Environmental Sciences, Nottingham Trent University, Brackenhurst Campus, Southwell, NG25 0QF, United Kingdom

**Keywords:** amino acid, broiler, metabolizable energy, oat hulls, particle size

## Abstract

Dietary fibres with increased particle size may develop foregut more efficiently in meat poultry fed diets moderately low in crude protein. The study investigated the performance of broilers fed low-density crude protein diets with the inclusion of oat hulls increasing in particle size. Ross 308 male broiler chicks (n = 336) were divided into 48 pens, 12 pens per treatment. Pens were allocated to 4 dietary treatments in mash form; positive control (**PC**), a standard crude protein diet, negative control (**NC**), around 5% lower in protein with 5% celite as an inert material, **OH400**: 5% lower protein diet with 5% oat hulls of geometric mean diameter (**GMD**) 400 µm, **OH850**: 5% lower protein diet with 5% oat hulls of GMD 850µm. Birds were fed ad libitum from d 1 to 35 in 3 phases; starter: d 0 to 10, grower: d 10 to 24 and finisher: d 24 to 35. Growth performance was calculated at the end of the trial. Two birds per pen were sampled on d 24 and 35 to collect data on proventriculus and gizzard weights and pooled ileal digesta. Apparent ileal digestibility of amino acids on d 24, and metabolizable energy on d 24 and 35 were recorded for each pen. Litter was sampled from each pen on d 34 to analyze litter N and moisture content. Footpad dermatitis scores of all birds per pen were recorded on d 35. Overall, no difference (*P* > 0.05) in body weight gain and feed intake was found between the treatments. However, NC and OH400 showed poorer FCR than PC, whereas FCR on PC and OH850 was similar (*P* > 0.05). Gizzard absolute weight and relative to body weight, and gizzard to proventriculus ratio were higher (*P* < 0.05) on OH850 compared to PC and NC on d 24 and 35. Gizzard digesta particle size was reduced (*P* < 0.05) on OH850 compared to all other diets on d 24 and 35. Amino acids digestibility coefficients for aspartic acid and valine increased (*P* < 0.05) in birds fed OH850 compared to PC, whereas coefficients for several other amino acids were improved compared to NC and OH400. The apparent ileal digestibility of metabolizable energy was similar (*P* > 0.05) between PC and OH850. Litter moisture and nitrogen, and footpad dermatitis scores were reduced (*P* < 0.05) on OH850 compared to PC. In conclusion, the inclusion of coarse oat hulls of GMD 850 µm in low-density crude protein diets can be beneficial for the broilers in developing the foregut, utilizing the nutrients efficiently and reducing litter nitrogen, moisture, and footpad scores.

## INTRODUCTION

Reducing the protein content of poultry diets has been cited as a route to reducing the environmental impact of animal protein production by lowering the excretion of nutrients, especially nitrogen, and improving the environmental footprint of the product (Greenhalgh et al., 2020). Previously published studies have shown that poultry growth performance has been impaired by the decrease of protein content in the diets when there are no additional amino acids added to the poultry diets (Kamran et al., 2008; Incharoen et al., 2010; Laudadio et al., 2012; Lemme et al., 2019). However, it is reported that supplementing additional amino acids regained reduced growth performance (Bennett and Classen, 2003; Ullrich et al., 2018; van Harn et al., 2019; Chrystal et al., 2020).

Adding fibres to poultry diets in moderate amounts improves gizzard function (Svihus, 2011), non-fibre nutrient digestibility (Jimenez-Moreno et al., 2009, 2010), gastrointestinal tract (**GIT**) health (Kalmendal et al., 2011; Mateos et al., 2012), and growth performance (Gonzalez-Alvarado et al., 2007; Jimenez-Moreno et al., 2013b). In addition, an adequate quantity of insoluble fibre in the diet, such as oat hulls (**OH**) and sunflower hulls, has been demonstrated to enhance digestion and performance in broiler chicks (Gonzalez-Alvarado et al., 2008; Svihus, 2011; Sacranie et al., 2012). Similarly, sugarcane bagasse, a by-product of sugarcane high in fibre content, has been shown to enhance the amount of Bacillus *spp*. bacteria in the intestine and, as a prebiotic, improve intestinal health and performance of broiler chickens (Kheravii et al., 2017). Fibre with larger particle sizes has been shown to improve gizzard function and intestinal development (Hetland et al., 2002). In this regard, consuming modest amounts of insoluble fibre or coarse particles in the diet increases the digesta retention time in the upper GIT, from the crop to the gizzard, which enhances the development and function of the gizzard (Nir et al., 1994; Hetland et al., 2005) and hence results in the improvement of the nutrient utilization (Svihus, 2011). By triggering the activity of cholecystokinin (Svihus et al., 2004), which arises in the duodenal and pyloric areas of the GIT in chicken, a well-developed gizzard promotes reverse peristalsis (Ferket, 2000; Denbow, 2015). It is reasonable to believe that insoluble fibre in digesta might take on a spongy structure, allowing enzymes to penetrate more easily (Sarikhan et al., 2010). As a result, the surface area and availability of nutrients to enzyme activity are enhanced, resulting in greater nutrient absorption and retention, as well as improved birds’ growth performance. Generally, the breakdown of fibre in chickens is believed to be insignificant, and its contribution to providing energy to the birds is considered minor (de Vries, 2015). However, fibre may have significant indirect effects on the nutritional value of chicken feed due to interactions with other nutrients. For instance, specific soluble fibre sources have been found to hinder the digestion of proteins, carbohydrates, and fats in the upper GIT (Choct and Annison, 1992; Smits et al., 1997). On the other hand, insoluble fibre sources like OH may improve the digestibility of nutrients (Sacranie et al., 2012; de Vries, 2015).

Recently, particle size has been reported to improve the performance of poultry by improving the foregut development which plays a vital role in the digestion and utilization of nutrients efficiently (Amerah et al., 2008; Zaefarian et al., 2016). The studies conducted on the particle size of structural components, for example, coarse or whole grains and fibres, evaluated the effect of particle size on poultry performance in standard protein diets where the impact of particle size on performance, gut development and nutrient utilization was the main objective of the studies. With the increased particle size of cereal grains or dietary fibres, the authors observed better weight gain, FCR, AMEn, and better nutrient utilization (Amerah et al., 2008; Donadelli et al., 2019). The increased feed particle size was found to be associated with increased digesta retention time in the upper GIT and exposure to gastric juices and digestive enzymes, resulting in improved nutrient utilization.

As low protein diets without additional amino acids result in lower growth rate, it is therefore quite reasonable to hypothesize that the lost growth performance of meat poultry due to the low protein content of the diets may be mitigated by increasing the digestive efficiency of the birds through the inclusion of structural fibre components. However, discerning the mechanism behind any effects of adding a fibre component to the diet is challenging due to the confounding effect of altered nutrient supply through the dilution introduced. Celite is an inert material sometimes used in poultry nutrition research as a marker for determining the digestibility of other nutrients. The inert and nonfibrous nature of celite means that it may be used to dilute a poultry diet without introducing any other alteration to the diet format, thus offering a negative control where diets are diluted without introducing structural fibre. Hence, a study was conducted to investigate the effect of large or small particle-size OH in low crude protein diets on the production performance, digestive organs, and nutrient utilization in broilers from day 1 to 35. The current study hypothesized that the deleterious effect of low protein diets on performance may be mitigated by increasing the digestive efficiency of the birds with the inclusion of dietary OH increasing in particle size rather than increasing their inclusion level, as well as improving the environmental footprint by lowering nitrogen excretion into the environment.

## MATERIALS AND METHODS

### Bird Husbandry

All experimental procedures involving animals were approved by the Nottingham Trent University's College of Science and Technology Ethical Review Committee (Approval No. ARE192063) and followed institutional and UK national NC3R ARRIVE guidelines for the care, use, and reporting of animals in research (Kilkenny et al., 2010).

A total of 336 male broiler chicks (Ross 308) were allocated to 48 pens with 7 chicks in each pen under a stocking density of 30 kg/m^2^. The experimental pens (floor area 0.64 m^2^) had bedding of softwood white shavings approximately 3 cm in depth. All the chicks had ad libitum access to feed and water throughout the experiment. On d 1, a 1-h dark period was provided then increased by an hour each day until reached 6 h, which was then maintained throughout the study. At chick placement, the temperature was set at 32°C and then dropped by approximately 0.5°C every day until it reached 21.5°C on d 21 then maintained through the rest of the experiment.

### Dietary Treatments

**Figure 1:**
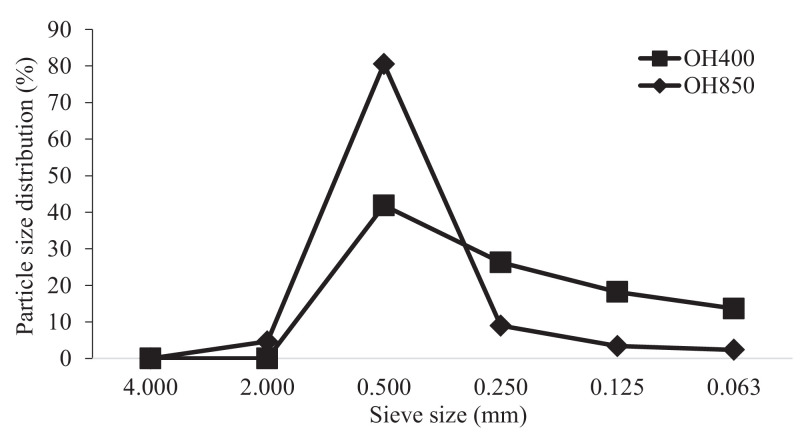
Particle size distribution (%) of oat hulls (OH) included in the diets.

Diets were provided in mash form to eliminate any confounding effect of pelleting on particle size and manufactured in-house before being assessed for gross energy (**GE**) by bomb calorimetry (Robbins and Firman, 2006). Oat hulls were ground using a cutting mill (Retsch GmbH, Germany) fitted with a 1.00mm screen for fine OH and a 3.75mm screen for coarse OH. Particle size distribution (%) of fine and coarse OH is given in Figure 1. Diets were formulated to meet the nutritional requirements of Ross 308 broilers. The standard commercial diet (PC) was diluted by adding 5% celite or OH (fine or coarse) for each growth phase of the birds, resulting in 4 dietary treatments for each phase of the growth period of birds as Starters diets (d 0–10); PC: commercial diet, CP:23.60%; NC: PC+5% celite, CP:22.41%; OH400: PC+5% Fine OH of geometric mean diameter (**GMD**): 400 µm, CP:22.61%; OH850: PC+5% Coarse OH of GMD: 850 µm, CP:22.61%; Grower diets (d 10–24); PC: commercial diet, CP:21.74%; NC: PC+5% celite, CP:20.65%; OH400: PC+5% Fine OH of GMD: 400 µm, CP:20.85%; OH850: PC+5% Coarse OH of GMD: 850 µm, CP:20.85%; Finisher diets (d 24-35); PC: Commercial diet, CP:20.05%; NC: PC+5% celite, CP:19.04%; OH400: PC+5% Fine OH of GMD: 400 µm, CP:19.24%; OH850: PC+5% Coarse OH of GMD: 850 µm, CP:19.24%.

To manufacture the diets, each feed ingredient was weighed individually and mixed dry for 5 min in a ribbon mixer (Rigal Bennett, Goole, Yorkshire, UK). After adding oil, the diets were further mixed for 5 min. At various stages, the mixer was brushed down to make sure that there were no oil clumps in the ribbon mixer. Each batch weight did not exceed 100kg for the appropriate mixing of the feed ingredients. Titanium dioxide (**TiO_2_**) as an inert marker was carefully included in each diet at 5 g/kg to ensure that digesta samples should have a sufficient amount of it for the determination of nutrients’ digestibility. Titanium dioxide (TiO_2_) was mixed with a dry mix before incorporation for homogeneity. Titanium dioxide (TiO_2_) level was analyzed for each diet to ensure that the diets were completely homogenized. The composition, amino acids and proximate analysis of diets are given in Tables 1 and 2.

**Table 1:** Diet composition (%) of starter (d 0–10), grower (d 10–24), and finisher (d 24–35).

Ingredients	Starter			Grower			Finisher		
	PC	NC	OH400^2^/OH850^3^	PC	NC	OH400/OH850	PC	NC	OH400/OH850
Wheat	57.20	54.29	54.29	61.55	58.43	58.43	65.35	62.04	62.04
Soybean meal	36.14	34.33	34.33	31.18	29.62	29.62	26.73	25.40	25.40
Soybean oil	2.54	2.42	2.42	3.50	3.32	3.32	4.51	4.28	4.28
Limestone	1.00	0.95	0.95	0.89	0.85	0.85	0.79	0.75	0.75
Salt	0.21	0.20	0.20	0.21	0.20	0.20	0.22	0.21	0.21
Sodium bicarbonate	0.16	0.15	0.15	0.17	0.16	0.16	0.17	0.16	0.16
Monocal phosphate	1.06	1.01	1.01	0.90	0.86	0.86	0.74	0.70	0.70
Lysine HCl	0.27	0.25	0.25	0.26	0.24	0.24	0.23	0.22	0.22
Methionine	0.33	0.31	0.31	0.29	0.27	0.27	0.25	0.24	0.24
Threonine	0.15	0.14	0.14	0.12	0.12	0.12	0.10	0.10	0.10
L-Valine	0.03	0.03	0.03	0.02	0.02	0.02	0.00	0.00	0.00
Celite-inert diluent	0.00	5.00	0.00	0.00	5.00	0.00	0.00	5.00	0.00
Oat hulls (fine or coarse)	0.00	0.00	5.00	0.00	0.00	5.00	0.00	0.00	5.00
Econase XT	0.01	0.01	0.01	0.01	0.01	0.01	0.01	0.01	0.01
Quantum Blue 5G	0.01	0.01	0.01	0.01	0.01	0.01	0.01	0.01	0.01
NTU Br. Premix^1^	0.40	0.40	0.40	0.40	0.40	0.40	0.40	0.40	0.40
Titanium dioxide	0.50	0.50	0.50	0.50	0.50	0.50	0.50	0.50	0.50

1 Premix content (volume/kg diet): Mn 100 mg, Zn 88 mg, Fe 20 mg, Cu 10 mg, I 1 mg, Mb 0.4 8mg, Se 0.2 mg, Retinol 13.5 mg, Cholecalciferol 3 mg, Tocopherol 25 mg, Menadione 5.0 mg, Thiamine 3 mg, Riboflavin 10.0 mg, Pantothenic acid 15 mg, Pyroxidine 3.0 mg, Niacin 60 mg, Cobalamin 30 µg, Folic acid 1.5 mg, Biotin 125 µg.

2 OH400: Oat hulls with GMD 400 µm.

3 OH850: Oat hulls with GMD 850 µm. Abbreviation: GMD, geometric mean diameter.

**Table 2:** Amino acid analysis (%) of grower diets and proximate analysis of the dietary treatments.

Diets	Parameter	PC	NC	OH400	OH850
Grower (d 10–24)	Cystine	0.36	0.34	0.37	0.34
	Aspartic acid	2.14	1.96	2.13	2.09
	Methionine	0.62	0.54	0.62	0.61
	Threonine	0.93	0.86	0.91	0.93
	Serine	1.14	1.04	1.06	1.08
	Glutamine	4.77	4.45	4.50	4.48
	Glycine	0.90	0.87	0.93	0.85
	Alanine	0.89	0.89	0.91	0.85
	Valine	1.00	0.99	1.03	0.96
	Isoleucine	0.93	0.86	0.92	0.90
	Leucine	1.66	1.50	1.56	1.59
	Tyrosine	0.52	0.37	0.45	0.47
	Phenylalanine	1.06	0.96	1.02	1.00
	Histidine	0.55	0.51	0.55	0.51
	Lysine	1.32	1.24	1.31	1.27
	Arginine	1.37	1.31	1.39	1.37
	Proline	1.33	1.19	1.22	1.30
Starter (d 0–10)	Dry matter (%)	89.00	89.00	89.00	89.00
	Ash (%)	6.09	10.89	6.00	5.38
	Crude protein (%)	23.60	22.41	22.61	22.61
	Gross energy (MJ/kg)	16.38	15.43	16.23	16.31
Grower (d 10–24)	Dry matter (%)	89.00	89.00	89.00	89.00
	Ash (%)	5.09	7.57	5.24	5.06
	Crude protein (%)	21.74	20.65	20.85	20.85
	Gross energy (MJ/kg)	16.21	15.38	16.36	16.23
Finisher (d 24–35)	Dry matter (%)	89.00	89.00	89.00	89.00
	Ash (%)	4.69	8.48	4.61	4.48
	Crude protein (%)	20.05	19.04	19.24	19.24
	Gross energy (MJ/kg)	16.53	15.74	16.70	16.52

Abbreviations: GMD, geometric mean diameter; PC, commercial diet, NC, PC+5% celite; OH400, PC+5% fine oat hulls of GMD 400 µm; OH850, PC+5% coarse oat hulls of GMD 850 µm.

### Growth Performance

Initial bird weight, final bird weight, bird weight gain, feed intake, and feed conversion ratio were recorded weekly. Mortality and weight of dead chickens were recorded daily.

### Bird Sampling, Digestive Organs, Litter Sampling, and Footpad Scores

Two representative birds, nearest to the pen's average weight, were sampled from each pen by cervical dislocation on d 24 and 35 of age. The empty weights of the proventriculus and gizzard were recorded and normalized for bird weight as g/kg body weight. The ileal digesta of each pen were pooled for the determination of amino acid digestibility on d 35 and apparent metabolizable energy (**AME**) digestibility on d 24 and 35. Gizzard digesta were pooled per pen for particle size determination on d 24 and 35. Ileal digesta samples were freeze-dried and finely ground and stored at room temperature (∼25°C) for further chemical analysis.

Litter samples were collected from the center and corners of each pen as described by Kamran et al. (2010) and then mixed to get 1 homogenous sample.

Footpad dermatitis (**FPD**) scoring was performed according to the guidelines of Aviagen Ltd., as described by de Jong and van Harn (2012). The scores of all birds were added up and the total score was used for data analysis.

### Particle Size Analysis

Particle size analysis for fine and coarse OH and freeze-dried gizzard digesta was performed by particle size distribution obtained through dry sieving using a vibratory shaker (Retsch, Germany) stacked with 6 sieves. The sieves were placed from top to bottom as 4.0 mm, 2.0 mm, 0.5 mm, 0.25 mm, 0.125 mm, and 0.063 mm, with a feed sample weight of 50 g, sieving time of 15 min, and amplitude of 3.0 mm/g. The GMD in µm and geometric standard deviation were calculated by the method described by Baker and Herrman (2002) as follows;

$$\text{Average}\;\text{Diameter}\;\text{of}\;\text{the}\;\text{two}\;\text{sieves}\left(d_{i}\right)={\left(d_{u}\times d_{o}\right)}^{1/2}$$

$$\text{Geometric}\;\text{Mean}\;\text{Diameter}\;\left(\text{GMD}\right)=\text{lo}g^{-1}\left[\sum \left(W_{i}\;\text{log}\;d_{i}\right)÷\sum W_{i}\right]$$

$$\text{Geometric}\;\text{Standard}\;\text{Deviation}\;\left(\text{GSD}\right)=\text{lo}g^{-1}{\left[\sum W_{i}{\left(\text{log}\;d_{i}-\text{log}\;d_{\text{gw}}\right)}^{2}÷\sum W_{i}\right]}^{1/2}$$

Where d_i_ is the diameter of i^th^ sieve in the stack, d_u_ is the diameter opening through which particles will pass (sieve proceeding i^th^), d_o_ is the diameter opening through which particles will not pass (i^th^ sieve), W_i_ is the weight of the sample retained on each sieve, d_gw_ is GMD.

### Chemical Analysis

Chemical analysis of feed samples or litter samples for dry matter (method 930.15), ash (method 942.05), and nitrogen (method 968.06) was performed by the official methods of the Association of Official Analytical Chemists (AOAC, 1990). Nitrogen content was determined by the combustion method (Dumas) using a nitrogen analyzer (Gerhardt, Germany). Crude protein content was calculated by multiplying nitrogen by a factor of 6.25. Titanium dioxide was administered at a rate of 5 g/kg in the diets as an inert marker for determining digestibility. Titanium dioxide concentration in diets and ileal digesta were determined as described by Short et al*.* (1996). A bomb calorimeter (Instrument 1261, Parr Instruments, IL) was used to determine the GE content of the feed and ileal digesta (Robbins and Firman, 2006). The AME was determined as follows;

$$\text{AME}\left(\text{MJ}/\text{kg}\right)=\text{Diet}\;\text{GE}-\;\left\{\text{Digesta}\;\text{GE}\times \left(\text{Diet}\;\text{Ti}O_{2}÷\text{Digesta}\;\text{Ti}O_{2}\right)\right\}$$

Where TiO_2_ is titanium dioxide as an inert marker in diet or digesta samples and GE is gross energy (MJ/kg).

Briefly, a Biochrom 30 amino acid analyzer (Biochrom Ltd., Cambridge, England) based on ion-exchange chromatography was used to determine the amino acid content of feed and digesta samples. Samples were prepared for analysis by oxidation with performic acid, followed by acid hydrolysis with 6N HCl and the addition of norleucine (2-aminohexanoic acid) as an internal standard. After internal standard correction, amino acid standards containing 200 nmol/mL of amino acids and norleucine were created and utilized to determine the amino acid content of the digesta and diets as described by Scholey et al*.* (2020). The apparent ileal digestibility coefficient of amino acids was calculated using the following equation:

AIDCoefficient=1−{(Nutrientindigesta×Tiindiet)÷(Nutrientindiet×Tiindigesta)}

Where Ti is a measured titanium concentration in either diet or digesta.

### Statistical Analysis

Analysis of variance was used to analyze data according to the following model;

Yij=M+TRTi+Bj+Eij

Where Y_ij_ is the response variable, M is the overall mean, TRT_i_ is the effect of dietary treatment, B_j_ is the effect of block and E_ij_ is the residual. Fisher's LSD method was used for mean comparison at *P* ≤ 0.05. Data analysis was carried out using the software JMP Pro14 (SAS Institute Inc., 2022).

## RESULTS

### Particle Size Analysis of Oat Hulls

The particle size distribution of OH included in the diets is given in Figure 1.

### Production Performance

For the overall experimental period (d 0–35), no difference (*P* > 0.05) in feed conversion ratio (**FCR**) was found between PC and OH850, however, diet PC showed better (*P* = 0.040) FCR than NC and OH400 (Table 3). There was no impact of dietary treatments on mortality and mortality was within normal ranges for the Nottingham Trent University Poultry Research Unit. Only 2 birds died in each group PC, OH400 and OH850 in the starter phase (d 0–10), and 2 birds died in OH400 in the grower phase (d 10–24). None of the birds died in the finisher phase (d 24–35).

**Table 3:** Effect of oat hulls varying in particle size in low-density crude protein diets on growth performance (d 0-35).

Parameter	Treatment^1^				SEM	*P*-value
	PC	NC	OH400	OH850		
Initial body weight (g)	44.04	44.18	44.31	44.06	0.240	0.843
Final body weight (g)	2145	2020	2026	2093	41.89	0.131
Body weight gain (g)	2101	1975	1981	2049	41.86	0.130
Feed intake (g)	3218	3162	3198	3243	50.17	0.716
Feed conversion ratio (FCR)	1.535^b^	1.605^a^	1.618^a^	1.584^ab^	0.021	0.040

1 Starters diets (d 0–10); PC: commercial diet, CP:23.60%; NC: PC+5% celite, CP:22.41%; OH400: PC+5% fine oat hulls of GMD: 400 µm, CP:22.61%; OH850: PC+5% coarse oat hulls of GMD: 850 µm, CP:22.61%. Grower diets (d 10–24); PC: Commercial diet, CP:21.74%; NC: PC+5% celite, CP:20.65%; OH400: PC+5% fine oat hulls of GMD: 400 µm, CP:20.85%; OH850: PC+5% coarse oat hulls of GMD: 850 µm, CP:20.85%. Finisher diets (d 24-35); PC: commercial diet, CP:20.05%; NC: PC+5% celite, CP:19.04%; OH400: PC+5% Fine oat hulls of GMD: 400 µm, CP:19.24%; OH850: PC+5% Coarse oat hulls of GMD: 850 µm, CP:19.24%.

ab Means within a row with different superscripts differ significantly (*P* ≤ 0.05).

### Absolute (G) and Relative Weights (G/Kg Body Weight) of Digestive Organs

The results, presented in Table 4, showed that gizzard absolute and relative weight (g per kg body weight), and gizzard to proventriculus ratio significantly increased on OH850 compared to PC, NC on d 24 (*P* < 0.001) and 35 (*P* = 0.003, 0.036 and 0.002, respectively). Gizzard digesta particle size as GMD decreased (*P* < 0.001) in birds fed OH850 compared to PC, NC and OH400 on d 24 and 35.

**Table 4:** Effect of oat hulls varying in particle size in low-density crude protein diets on digestive organ absolute weight (g) and relative weights (g/kg body weight) and gizzard digesta particle size.

Parameter	Treatment^1^				SEM	*P*-value
	PC	NC	OH400	OH850		
Day 24						
Gizzard absolute weight	21.67^b^	20.61^b^	21.20^b^	26.03^a^	0.732	<0.001
Gizzard relative weight	17.93^c^	20.02^b^	20.08^b^	24.26^a^	0.634	<0.001
Gizzard:proventriculus	3.882^b^	3.988^b^	4.167^b^	5.163^a^	0.131	<0.001
Day 35						
Gizzard absolute weight	29.18^b^	30.14^b^	30.12^b^	33.53^a^	0.813	0.003
Gizzard relative weight	13.72^b^	14.21^b^	14.75^ab^	16.01^a^	0.552	0.036
Gizzard:proventriculus	3.575^b^	3.832^b^	3.609^b^	4.325^a^	0.141	0.002
Gizzard digesta particle size						
Day 24						
GMD (µm)	1538^a^	1592^a^	1553^a^	1371^b^	33.89	<0.001
GSD	1.741	1.722	1.744	1.726	0.009	0.212
Day 35						
GMD (µm)	1525^a^	1532^a^	1572^a^	1333^b^	23.15	<0.001
GSD	1.737^b^	1.745^ab^	1.754^a^	1.721^c^	0.005	0.001

1 Starters diets (d 0–10); PC: commercial diet, CP:23.60%; NC: PC+5% celite, CP:22.41%; OH400: PC+5% fine oat hulls of GMD: 400 µm, CP:22.61%; OH850: PC+5% coarse oat hulls of GMD: 850 µm, CP:22.61%. Grower diets (d 10–24); PC: commercial diet, CP:21.74%; NC: PC+5% celite, CP:20.65%; OH400: PC+5% fine oat hulls of GMD: 400 µm, CP:20.85%; OH850: PC+5% coarse oat hulls of GMD: 850 µm, CP:20.85%. Finisher diets (d 24–35); PC: commercial diet, CP:20.05%; NC: PC+5% celite, CP:19.04%; OH400: PC+5% fine oat hulls of GMD: 400 µm, CP:19.24%; OH850: PC+5% coarse oat hulls of GMD: 850 µm, CP:19.24%.

abc Means within a row with different superscripts differ significantly (*P* ≤ 0.05). Abbreviations: GMD, geometric mean diameter; GSD, geometric standard deviation.

### Nutrient Utilization

Diet OH850 significantly increased (*P* < 0.05) the digestibility of aspartic acid and valine compared to diets PC, NC, and OH400. Digestibility coefficients for methionine, threonine, serine, isoleucine, leucine, tyrosine, phenylalanine, and proline were improved on diet OH850 compared to diets NC and OH400 but were similar (*P* > 0.05) to PC. AME digestibility was lower (*P* < 0.05) on OH400 compared to PC and OH850 on d 24 and 35 whereas no significant difference (*P* > 0.05) in the AME digestibility was observed between the diets PC and OH850 on d 24 and 35 (Table 5).

**Table 5:** Effect of oat hulls varying in particle size in low-density crude protein diets on apparent digestibility of amino acids and metabolizable energy.

Parameter	Treatment^1^				SEM	*P*-value
	PC	NC	OH400	OH850		
Ileal amino acid digestibility coefficients on d 24						
Cystine	0.741	0.707	0.718	0.737	0.013	0.254
Aspartic acid	0.798^b^	0.780^b^	0.791^b^	0.824^a^	0.009	0.007
Methionine	0.931^a^	0.912^b^	0.921^ab^	0.932^a^	0.004	0.002
Threonine	0.794^ab^	0.766^c^	0.771^bc^	0.820^a^	0.009	0.001
Serine	0.817^a^	0.788^b^	0.787^b^	0.829^a^	0.009	0.003
Glutamine	0.898	0.893	0.892	0.908	0.005	0.095
Glycine	0.776	0.758	0.764	0.789	0.009	0.108
Alanine	0.805	0.801	0.799	0.828	0.008	0.055
Valine	0.814^b^	0.806^b^	0.806^b^	0.836^a^	0.008	0.028
Isoleucine	0.822^ab^	0.807^b^	0.816^b^	0.843^a^	0.008	0.012
Leucine	0.839^ab^	0.823^b^	0.825^b^	0.858^a^	0.007	0.005
Tyrosine	0.863^a^	0.795^b^	0.826^b^	0.874^a^	0.013	<0.001
Phenylalanine	0.843^ab^	0.827^b^	0.833^b^	0.861^a^	0.007	0.010
Histidine	0.861	0.850	0.857	0.865	0.007	0.409
Lysine	0.879	0.871	0.875	0.890	0.005	0.055
Arginine	0.873	0.870	0.871	0.890	0.006	0.067
Proline	0.856^a^	0.832^b^	0.833^b^	0.873^a^	0.007	0.001
Ileal apparent metabolizable energy digestibility on d 24 and 35						
AME (MJ/kg) d 24	12.21^a^	11.29^bc^	10.86^c^	11.70^ab^	0.277	0.011
AME (MJ/kg) d 35	11.88^a^	11.75^ab^	11.39^b^	11.94^a^	0.137	0.035

1 Starters diets (d 0–10); PC: commercial diet, CP:23.60%; NC: PC+5% celite, CP:22.41%; OH400: PC+5% fine oat hulls of GMD: 400 µm, CP:22.61%; OH850: PC+5% coarse oat hulls of GMD: 850 µm, CP:22.61%. Grower diets (d 10–24); PC: commercial diet, CP:21.74%; NC: PC+5% celite, CP:20.65%; OH400: PC+5% fine oat hulls of GMD: 400 µm, CP:20.85%; OH850: PC+5% coarse oat hulls of GMD: 850 µm, CP:20.85%. Finisher diets (d 24-35); PC: commercial diet, CP:20.05%; NC: PC+5% celite, CP:19.04%; OH400: PC+5% fine oat hulls of GMD: 400 µm, CP:19.24%; OH850: PC+5% coarse oat hulls of GMD: 850 µm, CP:19.24%.

abc Means within a row with different superscripts differ significantly (*P* ≤ 0.05). Abbreviations: AME, apparent metabolizable energy; GMD, geometric mean diameter; MJ, megajoule.

### Litter Analysis and Footpad Scores

The results presented in Table 6, showed that OH850 decreased litter moisture (%) (*P* = 0.047) and nitrogen (%) (*P* < 0.0001) compared to diet PC on d 34. Footpad score was significantly reduced (*P* = 0.036) on the diet OH850 compared to PC on d 35.

**Table 6:** Effect of oat hulls varying in particle size in low-density crude protein diets on litter analysis (d 34) and footpad dermatitis score (d 35).

Attributes	Treatment^1^				SEM	*P*-value
	PC	NC	OH400	OH850		
Litter moisture (%)	33.03^a^	32.54^a^	29.87^ab^	24.10^b^	2.385	0.047
Litter N (%)	3.701^a^	3.168^c^	3.413^b^	3.281^bc^	0.068	<0.001
Footpad dermatitis scores^2^	0.250^a^	0.167^ab^	0.167^ab^	0.042^b^	0.067	0.036

1 Starters diets (d 0–10); PC: Commercial diet, CP:23.60%; NC: PC+5% celite, CP:22.41%; OH400: PC+5% fine oat hulls of GMD: 400 µm, CP:22.61%; OH850: PC+5% coarse oat hulls of GMD: 850 µm, CP:22.61%. Grower diets (d 10-24); PC: commercial diet, CP:21.74%; NC: PC+5% celite, CP:20.65%; OH400: PC+5% fine oat hulls of GMD: 400 µm, CP:20.85%; OH850: PC+5% Coarse oat hulls of GMD: 850 µm, CP:20.85%. Finisher diets (d 24–35); PC: commercial diet, CP:20.05%; NC: PC+5% celite, CP:19.04%; OH400: PC+5% fine oat hulls of GMD: 400 µm, CP:19.24%; OH850: PC+5% coarse oat hulls of GMD: 850 µm, CP:19.24%.

2 Footpad dermatitis scores per pen: footpad score of bird 1 + footpad score of bird 2 + footpad score of bird 3 + footpad score of bird 4 + footpad score of bird 5.

abc Means within a row with different superscripts differ significantly (*P* ≤ 0.05).

## DISCUSSION

This study aimed to determine whether a moderate inclusion of OH increasing in particle size, could be introduced to develop moderately low-protein diets and improve digestive efficiency via improved GIT development. Oat hulls, a by-product of the oat grains, are high in fibre content and can be used to dilute the protein content of poultry diets which may be economical and improve the digestion of moderate low protein diets.

The study showed no significant difference in body weight gain and feed intake between the treatments for the whole study period (d 0–35). However, the feed conversion ratio was poor on reduced crude protein diets with inert material celite (NC) and the fine OH diet (OH400). The diet with coarse OH (OH850) was comparable to a standard protein diet (PC) in terms of feed conversion ratio which may be the result of better gizzard development. This can be supported by the increased gizzard absolute weight (g) and relative weight (g/kg BW) on d 24 and 35 which was significantly higher on the OH850 compared to PC and NC. The growth performance results were partly in agreement with the findings of Jimenez-Moreno et al*.* (2010) as they found that body weight gain, feed intake and FCR were not affected by the particle size of OH included in the diets. However, the current study found a positive effect on FCR due to the inclusion of coarse OH in low-protein diets. This difference may be due to the varying particle sizes of OH used in their study (fine:386 µm vs. coarse: 462 µm) compared to the current one. Hetland and Svihus (2001) discovered that the feed conversion ratio was poorer in young broilers fed diets containing 10% coarsely ground OH in contrast to birds fed 10% finely ground OH. However, the current study observed better FCR on coarse OH inclusion in low-protein diets. The reason for the disagreement with those authors on the response of broilers to FCR may be the inclusion rate of OH as, in the current study, only 5% of OH were employed vs. 10% in that study.

Coarse OH inclusion, in the current study, might have promoted the grinding activity of the gizzard allowing for better development of the muscular layers and an increase in organ size (Rogel et al., 1987; Gonzalez-Alvarado et al., 2008). On d 24 and 35, the gizzard digesta particle size was reduced on the OH850 diet compared to the other diets. The lower particle size of gizzard digesta on the low protein diet with the coarse hull (OH850) compared with the standard protein diet (PC) may suggest the increased grinding activity of the gizzard which is reflected in the reduced particle size of gizzard digesta, though the diet OH850 had the largest particle size due to inclusion of coarse OH. The gizzard-to-proventriculus ratio was significantly increased on OH850 compared to PC, NC, and OH400 at both age points d 24 and 35 in the current study. The higher gizzard-to-proventriculus ratio indicates a remarkable difference in gizzard and proventriculus rather than just 1 compartment of gizzard and proventriculus. A poorly developed gizzard results in lower gizzard-to-proventriculus which gives rise to dilatation of proventriculus and both the stomach parts act as transit organs instead of grinding and mixing of gastric juices. The dilation of the proventriculus, a condition where the proventriculus gets dilated and cannot be well demarked from gizzard, is condemned by the meat processing industry as this contaminates the processing line (Amer, 2021). These findings were in agreement with the finding of Taylor and Jones (2004). These authors explained that feeding coarsely ground diets to poultry can reduce the proventricular dilatation and result in better mixing and grinding of the feed material in the upper digestive tract.

Amino acid digestibility of aspartic acid and valine was improved significantly on OH850 compared to a standard protein diet, diet diluted with either celite or fine OH, whereas digestibility of threonine, serine, isoleucine, leucine, tyrosine, phenylalanine, and proline was significantly increased on coarse hull diet compared to low protein diet either with celite or fine oat hull but was comparable to the standard diet. This can be explained by that the increased particle size of OH caused an increase in gizzard activity, more gastric juice and hydrochloric acid are secreted to breakdown the peptide bond of the amino acids before they pass to the hindgut where they are absorbed resulting in increased digestibility (Ravindran and Abdollahi, 2021). Increased grinding activity is also reflected by the significantly reduced particle size of gizzard digesta on d 24 and 35 on the coarse oat hull diet in the current study. Additionally, this effect may be due to OH’ ability to stimulate: 1) the gizzard to improve its development and function (Gonzalez-Alvarado et al., 2008; Sacranie et al., 2012); and 2) the gut reflex and enzyme production to improve nutrient digestibility (Svihus, 2011; Jimenez-Moreno et al., 2013a). In the current study, birds fed OH850 had higher ileal AME on d 24 and 35 than those fed OH400. Indeed, a large, well-developed gizzard improves gut motility (Ferket, 2000) by increasing the release of cholecystokinin (Svihus et al., 2004), which occurs in the duodenum and pylorus of fowls (Denbow, 2015) and acts via the vagus nerve to stimulate pancreatic enzyme secretion and gastroduodenal reflux (Duke, 1992; Li and Owyang, 1993). Coarse particles improve the exposure time of nutrients to digestive enzymes by delaying the transit time of digesta in the gizzard (Nir et al., 1994), hence enhancing energy consumption and nutritional digestibility. Furthermore, a lower pH in the gizzard has been shown to stimulate pepsin activity (Gabriel et al., 2003), which accelerates the denaturing and hydrolysis of food proteins and hence improves protein digestion. Several studies have found that feeding structural materials; coarse or whole grain and fibres, to birds, improve nutrient utilization (Svihus and Hetland, 2001; Rougiere et al., 2009). A faster feed transit in the digestive tract has been proposed to limit the amount of time available for digestion (Hetland and Svihus, 2001). However, the period available for microbial fermentation in the small intestine reduces, perhaps making more substrate available to the bird. When OH are incorporated into wheat diets, starch digestibility increases (Hetland and Svihus, 2001) which may be the factor for improved AME digestibility in the current study on reduced crude protein diets with the inclusion of coarse OH. When coarse OH were utilized, the improvement in starch digestibility was greater showing that the favorable impact of OH is connected to the effects of coarse particles (Rogel et al., 1987). The reason for this comparable AME digestibility to the standard protein diet is unknown; however, it might be linked to an increase in gizzard activity, which stimulates pancreatic secretion. Additionally, Hetland et al. (2003) observed a considerable increase in amylase activity and bile acid output which may be used for enhanced lipid digestibility with the inclusion of OH.

The current study results showed that the diet moderately low in protein with coarse OH significantly reduced litter moisture and nitrogen compared with the standard diet, PC. Controlling litter moisture and nitrogen content is important to maintain productivity and avoid environmental and bird welfare issues. High crude protein content in diets has been associated with high water intake and high excretion of water through urination (James and Wheeler, 1949; Hilliar et al., 2020), and higher nitrogen excretion (Lemme et al., 2019). In the current study, the low litter moisture content on a coarse hull diet may be linked to lower ammonia emissions and fewer welfare issues, such as hock burns and foot pad dermatitis (Miles et al., 2011). There are a few plausible explanations for how oat hull diets can reduce water excretion. To begin, fibre can contain a huge quantity of water in its matrix, and this capacity varies depending on the type of fibre employed. A longer digesta retention time combined with a higher water holding capacity in the gut would enhance water reuse in the caeca and minimize water excretion. Second, the gizzard of birds given a dietary fibre diet would operate as a pacemaker organ for nutrient digestion and absorption, regulating water absorption to an optimal level which would lead to low water quantity excreted in the litter by reducing the birds’ unneeded desire to drink excessively. Footpad dermatitis scores were significantly reduced on the coarse oat hull diet compared with the standard diet, FPD is another welfare issue in the modern broiler industry which has been recognized in Europe and North America for several decades, and its frequency is remarkably high in floor-housed broilers and turkeys (Mayne, 2005; Shepherd and Fairchild, 2010). Recently, this problem has gained prominence as an essential indication of animal welfare (Bessei, 2006). A significantly lower score of FPD on the coarse oat hull diet may be explained by the lower moisture content of the litter in coarse oat hull diets in the current study (Taira et al., 2014).

In conclusion, including 5% coarse OH and reducing crude protein of poultry diets by around 5% did not compromise the growth performance of broilers. Moreover, these diets improved nutrient utilization and bird welfare parameters and decreased the environmental impact of broilers in terms of lower nitrogen excretion. Improved nutrient utilization could be attributed to a well-developed gizzard as a result of feeding the broilers diets containing coarsely ground OH. This might be the most likely one of the underlying causes of the improved performance and nutrient utilization and better litter and welfare on low-density crude protein diets seen in this study. However, celite is diatomaceous earth and is high in ash content. In the NC group, it might have impacted the digestibility of amino acids due to having no nutritional or structural value compared to OH, or intestinal microbial ecology, which needs further investigation. Developing low-protein diets with the inclusion of coarse OH of GMD 850µm have the potential to decrease environmental pollution and feeding cost. OH, a byproduct of oat cereals can be used in manufacturing poultry diets with a moderate reduction in crude protein content contributing to the sustainability of protein resources. Coarse grinding of OH can increase the digestive tract efficiency by developing the foregut more efficiently. Further research on the gradual reduction in the crude protein content of poultry diets with a gradual increase of coarse OH may benefit the birds by utilizing them efficiently and optimally with the increased digestive efficiency, leading to a reduced environmental footprint and sustainable meat production.
